# High-resolution image-based simulation reveals membrane strain concentration on osteocyte processes caused by tethering elements

**DOI:** 10.1007/s10237-021-01511-y

**Published:** 2021-09-01

**Authors:** Yuka Yokoyama, Yoshitaka Kameo, Hiroshi Kamioka, Taiji Adachi

**Affiliations:** 1 Department of Micro Engineering, Graduate School of Engineering, Kyoto University, 53 Shogoin-Kawahara-cho, Sakyo-ku, Kyoto, 606-8507 Japan; 2 Department of Biosystems Science, Institute for Frontier Life and Medical Sciences, Kyoto University, 53 Shogoin-Kawahara-cho, Sakyo-ku, Kyoto, 606-8507 Japan; 3 Department of Mammalian Regulatory Network, Graduate School of Biostudies, Kyoto University, 53 Shogoin-Kawahara-cho, Sakyo-ku, Kyoto, 606-8507 Japan; 4 Department of Orthodontics, Graduate School of Medicine, Dentistry and Pharmaceutical Sciences, Okayama University, 2-5-1 Shikata-Cho, Kita-ku, Okayama, 700-8525 Japan

**Keywords:** Osteocyte, Mechanosensing, Canaliculus, Tethering element, Image-based simulation, Computational biomechanics

## Abstract

Osteocytes are vital for regulating bone remodeling by sensing the flow-induced mechanical stimuli applied to their cell processes. In this mechanosensing mechanism, tethering elements (TEs) connecting the osteocyte process with the canalicular wall potentially amplify the strain on the osteocyte processes. The ultrastructure of the osteocyte processes and canaliculi can be visualized at a nanometer scale using high-resolution imaging via ultra-high voltage electron microscopy (UHVEM). Moreover, the irregular shapes of the osteocyte processes and the canaliculi, including the TEs in the canalicular space, should considerably influence the mechanical stimuli applied to the osteocytes. This study aims to characterize the roles of the ultrastructure of osteocyte processes and canaliculi in the mechanism of osteocyte mechanosensing. Thus, we constructed a high-resolution image-based model of an osteocyte process and a canaliculus using UHVEM tomography and investigated the distribution and magnitude of flow-induced local strain on the osteocyte process by performing fluid–structure interaction simulation. The analysis results reveal that local strain concentration in the osteocyte process was induced by a small number of TEs with high tension, which were inclined depending on the irregular shapes of osteocyte processes and canaliculi. Therefore, this study could provide meaningful insights into the effect of ultrastructure of osteocyte processes and canaliculi on the osteocyte mechanosensing mechanism.

## Introduction

Osteocytes are the most abundant cells present inside the bone matrix and are vital for bone remodeling as they regulate osteoclastic bone resorption and osteoblastic bone formation in response to a mechanical stimuli (Tatsumi et al. 2007; Nakashima et al. 2011; Xiong et al. 2011). Typically, osteocytes are sensitive to local mechanical stimuli, especially in cell processes (Adachi et al. 2009; Wu et al. 2011). Results of prior research have indicated that a reduction in mechanical stimuli owing to disuse causes osteoporotic conditions in the bone structure (Aguirre et al. 2006; Liu et al. 2020). Therefore, the clarification of mechanosensing mechanisms related to osteocyte processes is necessary for understanding the cellular contribution toward maintaining the physiological bone structure.

In the mechanosensing of osteocytes, local strain on the cell process membrane is considered a representative indicator of mechanical stimuli as it can activate gating of mechanosensitive ion channels such as Piezo1 (McMahon et al. 2008; Li et al. 2019; Diem et al. 2020; Sasaki et al. 2020; Zhou et al. 2020). Although a ~ 10% strain on the bone cell membranes can induce Ca^2+^ responses (You et al. 2000), the strain of mineralized bone tissue during normal locomotion does not exceed ~ 0.1% (Fritton et al. 2000). Thus, a strain amplification model was proposed in previous studies to infuse the gap in strain magnitude between the cellular and tissue levels (Weinbaum et al. 1994; Zeng et al. 1994; You et al. 2001; Han et al. 2004; Wang et al. 2007, 2014; Scheiner et al. 2016). In context, the interstitial fluid flow generated by external forces in the canalicular space is considered a principal factor in strain amplification (Weinbaum et al. 1994; Zeng et al. 1994; Scheiner et al. 2016). Furthermore, the pericellular matrix in the canalicular space, such as proteoglycans, can additionally facilitate strain amplification. Specifically, tethering elements (TEs) connecting the osteocyte process to the canalicular wall, which are regarded as the core protein of perlecan, are supposed to provide drag force to the cell processes via interstitial fluid flow (You et al. 2001; Han et al. 2004; Wang et al. 2007, 2014).

The impacts of interstitial fluid flow and TEs on the strain of osteocyte processes have been revealed through theoretical analyses (You et al. 2001; Han et al. 2004; Wang et al. 2007), in which the mechanism of strain amplification via fluid flow and TEs was clarified using idealized cylindrical models representing the osteocyte processes and canaliculi. Despite the significance of this mechanism, the irregular shape of osteocyte processes and canaliculi is important for amplifying the strain as it can induce heterogeneous distribution of interstitial fluid velocity (Kamioka et al. 2012) and thus affect the local strain on the cell processes. Furthermore, the deterioration in the canalicular structure owing to aging or bone diseases (Jast and Jasiuk 2013; Lai et al. 2015; Tiede-Lewis and Dallas 2019) can alter the flow-induced strain of osteocytes and influence subsequent bone remodeling. Therefore, the contribution of the irregular shape of osteocyte processes and canaliculi toward influencing the local osteocyte strain is required to be investigated.

Computational studies utilizing image-based models are useful for quantitatively evaluating the mechanical stimuli required for osteocyte processes. In previous studies, the lacuno-canalicular model constructed from confocal laser scanning images was used to investigate the distribution of interstitial fluid velocity and osteocyte strain (Verbruggen et al. 2012, 2014, 2016). Although the confocal laser scanning microscopy cannot appropriately capture the microscopic surface of osteocyte processes and canaliculi, ultra-high voltage electron microscope (UHVEM) tomography enables observations of their ultrastructure at a nanometer scale (Kamioka et al. 2012; Hosaki-Takamiya et al. 2016). Moreover, computational analysis using such high-resolution image-based models can potentially reveal the influence of the ultrastructure on the local strain of osteocyte processes.

This study aims to characterize the roles of the ultrastructure of osteocyte processes and canaliculi in the osteocyte mechanosensing mechanism via interstitial fluid flow and TEs. Thus, we constructed a high-resolution image-based model of an osteocyte process and a canaliculus using UHVEM tomography (Kamioka et al. 2012). The influence of TEs on the mechanical stimuli for osteocyte processes was investigated by introducing randomly distributed spring models inside the canalicular space of the image-based model. Subsequently, the distribution and magnitude of the flow-induced local strain on the osteocyte process were examined through fluid–structure interaction simulation, the results of which revealed that the heterogeneous inclination of TEs produced by the irregular shape of osteocyte processes and canaliculi is a key factor in activating osteocyte mechanosensing.

## Methods

### Fluid–structure interaction simulation

The deformation of osteocyte processes induced by interstitial fluid flow and TEs was investigated by developing a method for fluid–structure interaction simulation based on previous studies (Takeishi et al. 2019). In addition, interstitial fluid flow through the pericellular matrix in the canaliculus was assumed to follow the Brinkman equation (Weinbaum et al. 1994), in which the fluid velocity, ***u***, induced by the pressure gradient, $\nabla p$, and the body force, ***F***, can be described as

1 $$\nabla p = - \frac{\mu }{{k_{{\text{p}}} }}{\varvec{u}} + \mu \nabla^{2} {\varvec{u}} + {\varvec{F}},$$

where *μ* is the fluid viscosity and *k*_p_ the permeability of the pericellular matrix. Furthermore, the lattice Boltzmann method (Chen and Doolen 1998) was employed for the numerical analysis of the fluid flow governed by the Brinkman equation.

The osteocyte process membrane was modeled as a thin hyperelastic membrane and discretized into triangular finite elements. The deformation of the hyperelastic membrane was assumed to be governed by Skalak’s constitutive law (Skalak et al. 1973), in which the strain energy function can be expressed as

2 $$W = \frac{1}{4}G_{{\text{s}}} \left( {I_{1}^{2} + 2I_{1} - 2I_{2} + CI_{2}^{2} } \right),$$

where *I*_1_ and *I*_2_ are the first and second strain invariants of the right Cauchy–Green tensor, $G_{{\text{s}}}$ the surface shear elastic modulus, and *C* the area incompressibility coefficient. The surface Poisson’s ratio, $\nu_{{\text{s}}}$, and Young’s modulus, *E*, of the osteocyte cell membrane can be expressed using the membrane thickness, *t*, as $\nu_{{\text{s}}} =$ $\frac{C}{C + 1}$ and $E =$ $\frac{{2G_{{\text{s}}} \left( {1 + \nu_{{\text{s}}} } \right)}}{t}$. In addition, the restoring force, ***q***_m_, on the membrane node, ***x***_m_, was calculated from the membrane displacement based on a finite element procedure. Here, all the displacements of the membrane nodes were determined from the advection of the fluid, as described next.

A TE was introduced as a linear elastic spring, which connects a point on the membrane of the osteocyte process, ***x***_m_, to a point on the canalicular wall, ***x***_w_. The point of attachment of each TE on the canalicular wall, ***x***_w_, was constant during the simulation. The spring constant, $k_{{{\text{sp}}}}$, was determined as $k_{{{\text{sp}}}} =$ $\frac{{\pi r_{{{\text{sp}}}}^{2} E_{{{\text{sp}}}} }}{{l_{0} }}$, where $E_{{{\text{sp}}}}$, $r_{{{\text{sp}}}}$*,* and $l_{0}$ are the Young’s modulus, radius, and the natural length of TEs, respectively. Consequently, the restoring force, ***q***_t_, acting on the membrane node induced by the stretching of the TEs, i.e. the displacement of the membrane node ***x***_m_, was calculated as

3 $${\varvec{q}}_{{\text{t}}} \left( {{\varvec{x}}_{{\text{m}}} } \right) = \left\{ {\begin{array}{*{20}l} { - k_{{{\text{sp}}}} \left( {\left| {{\varvec{x}}_{{\text{m}}} - {\varvec{x}}_{{\text{w}}} } \right| - l_{0} } \right)\frac{{{\varvec{x}}_{{\text{m}}} - {\varvec{x}}_{{\text{w}}} }}{{\left| {{\varvec{x}}_{{\text{m}}} - {\varvec{x}}_{{\text{w}}} } \right|}}} \hfill & {\left( {\left| {{\varvec{x}}_{{\text{m}}} - {\varvec{x}}_{{\text{w}}} } \right| > l_{0} } \right)} \hfill \\ {\varvec{0}} \hfill & {\left( {\left| {{\varvec{x}}_{{\text{m}}} - {\varvec{x}}_{{\text{w}}} } \right| \le l_{0} } \right)} \hfill \\ \end{array} } \right..$$

Moreover, the immersed boundary method (Peskin 2002) was employed to couple the interstitial fluid flow and cell membrane deformation. The total restoring force acting on the membrane, ***q*** = ***q***_m_ + ***q***_t_, was distributed to the surrounding fluid nodes, ***x***_f_, and it acted as an external body force, ***F***, expressed as

4 $${\varvec{F}}\left( {{\varvec{x}}_{{\text{f}}} } \right) = \sum D\left( {{\varvec{x}}_{{\text{f}}} - {\varvec{x}}_{{\text{m}}} } \right){\varvec{q}}\left( {{\varvec{x}}_{{\text{m}}} } \right),$$

where *D*(***x***) is the numerically approximated Dirac delta function given by

5 $$D\left( {\varvec{x}} \right) = \left\{ {\begin{array}{*{20}l} {\frac{1}{{64\Delta x^{3} }}\prod\limits_{i = 1}^{3} {\left( {1 + \cos \frac{{\pi x_{i} }}{2\Delta x}} \right)} } \hfill & {{\text{for}}\;\left| {x_{i} } \right| \le 2\Delta x,\;\;i = 1,2,3} \hfill \\ 0 \hfill & {{\text{otherwise}},} \hfill \\ \end{array} } \right.$$

where $\Delta x$ is the lattice interval in the fluid domain, which is also described in Sect. 2.2. Conversely, the cell membrane was advected with velocity ***U*** obtained by interpolating the surrounding fluid velocity, ***u***, using the approximated Dirac delta function as

6 $${\varvec{U}}\left( {{\varvec{x}}_{{\text{m}}} } \right) = \sum D\left( {{\varvec{x}}_{{\text{m}}} - {\varvec{x}}_{{\text{f}}} } \right){\varvec{u}}\left( {{\varvec{x}}_{{\text{f}}} } \right)\Delta x^{3} ,$$

to satisfy the non-slip condition on the membrane surface. By using the displacement of membrane node ***x***_m_ advected by membrane velocity ***U***, restoring force ***q***_m_ on the membrane node was calculated again.

### Reconstruction of image-based models

**Fig. 1:**
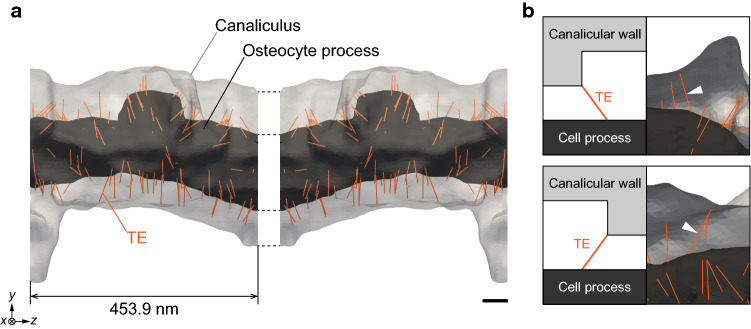
**a** Reconstruction of an image-based model of osteocyte process and canaliculus. TEs connecting osteocyte process membrane and canalicular wall were randomly placed in the canalicular space (*n* = 5). Scale bar = 50 nm. **b** Inclined TEs in the image-based model. Inclination of TEs depends on the surface shape of canalicular wall, as indicated by the arrow head

The three-dimensional image-based model of an osteocyte process and surrounding canaliculus (Fig. 1a) was reconstructed from UHVEM tomographic images (Kamioka et al. 2012), and a Cartesian coordinate system (*x*, *y*, *z*) was set along the longitudinal direction of the osteocyte process with the canaliculus in the *z* direction. The original resolution of the tomographic images was 1.7 nm/pixel, and the length of the cell process and canaliculus along the *z* direction was 453.9 nm.

Based on these images, the canalicular space was discretized by a cubic lattice with intervals $\Delta x =$ 5.1 nm (= 3 pixels) for the lattice Boltzmann method. Thereafter, the hyperelastic membrane representing the osteocyte process membrane was discretized into triangular finite elements using MeshLab (Visual Computing Lab, ISTI‐CNR, Italy). Typically, the representative length of the individual elements was ~ 4.7 nm. The attachment points of TEs on the osteocyte process were randomly arranged with a number density of 12/37.5 nm^−1^ along the *z* direction (Han et al. 2004; You et al. 2004; Wang et al. 2007). Then, the other end of each TE was attached to the nearest point on the canalicular wall. As each TE was inclined under no-flow conditions depending on the irregular shape of the canalicular wall (Fig. 1b), its natural length, $l_{0}$, was set as the length under no-flow condition. In this study, we constructed five models, each of which has a different random configuration of TEs. Furthermore, a mirror symmetric model was connected to the original model with respect to the *x*–*y* plane to apply the periodic boundary conditions for the fluid–structure interaction simulation, as depicted in Fig. 1a.

The values of the parameters used in the simulation are listed in Table 1 (Weinbaum et al. 1994; Sugawara et al. 2008; Kamioka et al. 2012; Takeishi et al. 2014; Wijeratne et al. 2016). Thus, the local strain on the osteocyte process was simulated under reciprocal fluid flow along the positive/negative *z* direction (hereinafter called + *z*/− *z* flow) with a pressure gradient of $\left| {\nabla p} \right| = 1.0\;{\text{Pa/nm}}$ (Kamioka et al. 2012) along the *z* direction.

**Table 1:** Parameter settings for the fluid–structure interaction simulation (Weinbaum et al. 1994; Sugawara et al. 2008; Kamioka et al. 2012; Takeishi et al. 2014; Wijeratne et al. 2016)

Symbol (unit)	Description	Value
*Fluid properties*		
*μ* (Ns/m^2^)	Fluid viscosity	1.0 × 10^−3a^
*k*_p_ (nm^2^)	Permeability of PCM	7.0^b^
*Membrane properties*		
*C*	Area incompressibility coefficient	10^c^
*E* (Pa)	Young’s modulus of membrane	4471^d^
*t* (nm)	Thickness of membrane	10^e^
*TE properties*		
*E*_sp_ (MPa)	Young’s modulus of TEs	71^f^
*r*_sp_ (nm)	Radius of TEs	1^f^

^a^Kamioka et al. (2012)

^b^Weinbaum et al. (1994)

^c^In reference to the value of red blood cells (Takeishi et al. 2014)

^d^Sugawara et al. (2008)

^e^In reference to the thickness of lipid bilayer

^f^Wijeratne et al. (2016)

## Results

**Fig. 2:**
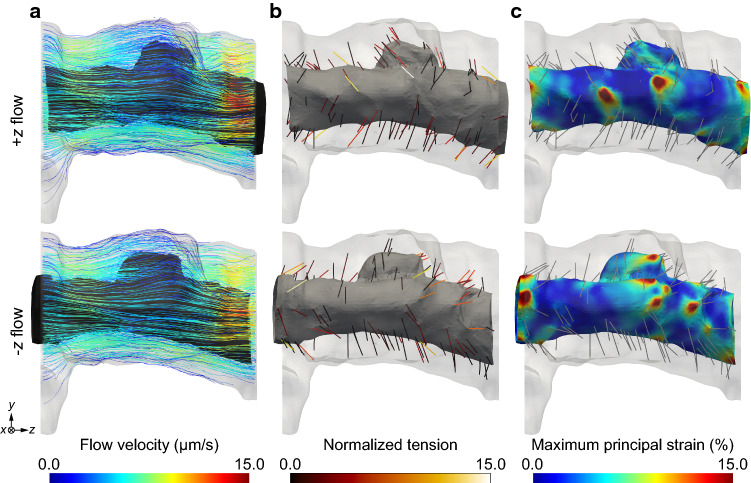
Fluid–structure interaction simulation under + *z*/− *z* flow conditions. **a** Flow velocity of interstitial fluid. **b** Tension of TEs normalized by its median (0.017 pN). **c** Maximum principal strain on osteocyte process

The image-based fluid–structure interaction simulation reflected the flow-induced strain on the osteocyte process (Fig. 2). Although the distributions of flow velocity magnitude under + *z* and − *z* flow conditions exhibited slight variations (Fig. 2a), the distributions of TEs with high tension (Fig. 2b) and those of the maximum principal strain on the osteocyte process (Fig. 2c) were significantly dependent on the flow direction. Moreover, the strain concentration was detected in the neighborhood of the TEs with higher tension (Fig. 2b, c). These observations were common in the five models with varying random TE configurations (data not presented).

**Fig. 3:**
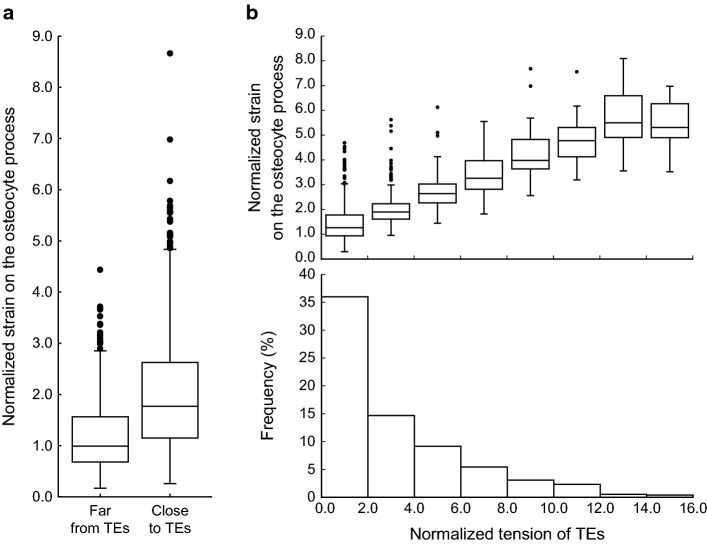
Relationship between strain on osteocyte process and tension of TEs. **a** Osteocyte process strain on the regions within 10.2 nm of TEs and on other regions far from the TEs. Strain is normalized by its median far from the TEs (0.028). **b** Osteocyte process strain on regions near TEs with varying tensions and histogram of TEs with nonzero tension. Tension is normalized by its median (0.017 pN)

Quantitative analysis results of the osteocyte process strain revealed that the strain in the vicinity of the TEs was 1.7 times larger than that present farther from the TEs (Fig. 3a). Here, we defined the vicinity of the TEs as the area within 10.2 nm from each TE, which is equivalent to twice of the fluid lattice interval and a quarter of the representative length of TE interval on the cell process membrane. Moreover, this larger local strain was caused by TEs with higher tension (Fig. 3b top). Interestingly, the percentage of TEs with high tension, primarily contributing to the generation of a large local strain, was extremely small (Fig. 3b bottom), indicating that a small number of TEs with high tension are responsible for supporting the osteocyte process and inducing a strain concentration in the cell process.

**Fig. 4:**
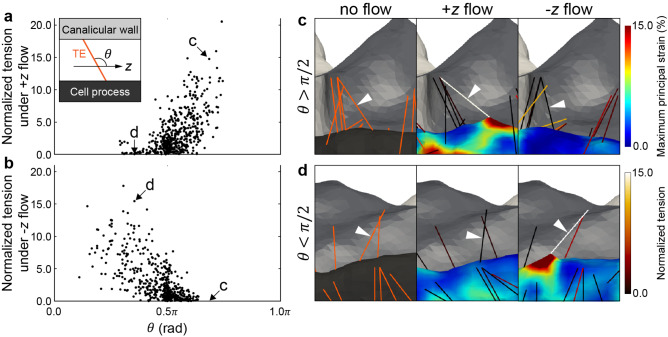
Dependence of tension in TEs and membrane strain of osteocyte process on inclination of TEs. **a**, **b** Tension of TEs under **a** + *z* flow and **b** − *z* flow plotted against the orientation angle under no-flow condition *θ* from the positive *z* direction. **c**, **d** Mechanical behavior of **c** TE with $\theta > \pi {/}2$ (arrow head) and **d** TE with $\theta < \pi {/}2$ (arrow head) under no-flow, + *z* flow, and − *z* flow conditions. Osteocyte process strain and TEs tension are also depicted. Tension is normalized by its median (0.017 pN)

The TEs with high tension that produce a strain concentration on the osteocyte process were identified by plotting the tension of TEs against their orientation angle under no-flow condition $\theta$ from the positive *z* direction (Fig. 4). As shown in Fig. 4a, TEs with a large value of $\theta$ under the + *z* flow condition—TEs aligned upstream on the osteocyte process—produced high tension. On the contrary, TEs with a small $\theta$ under the − *z* flow condition generated high tension (Fig. 4b). Upon focusing on individual TEs, the TE that was aligned upstream in the + *z* flow ($\theta > \pi {/}2$) generated a strain concentration on the osteocyte process under the + *z* flow condition (Fig. 4c), whereas the TE inclined upstream to the − *z* flow ($\theta < \pi {/}2$) generated a strain concentration under the − *z* flow condition (Fig. 4d). These results indicate that the relative angle of the TEs to the flow direction, which can be determined based on the irregular shapes of osteocyte processes and canaliculi, is a crucial factor in producing a strain concentration in the osteocyte process.

## Discussion

The influences of the ultrastructure of osteocyte processes and canaliculi on the mechanical stimuli to osteocytes were investigated by evaluating the flow-induced strain on the osteocyte processes through image-based fluid–structure interaction simulation (Fig. 1). Consequently, the simulation results revealed that specific TEs with high tension, which depend on the irregular shapes of osteocyte processes and canaliculi, can generate a local strain concentration in the osteocyte process. The local large strain induced by such TEs can potentially activate mechanical responses of osteocytes by causing structural changes in ion channels on the neighboring cell membrane (Hayakawa et al. 2008). Therefore, the strain concentration on the osteocyte process via TEs is a vital mechanism that is responsible for osteocyte mechanosensing.

Previous studies have theoretically proposed the mechanism of strain amplification in osteocyte mechanosensing, in which the fluid drag on the pericellular matrix induces a large strain on the cell process via TEs (You et al. 2001; Han et al. 2004; Wang et al. 2007). Although this strain amplification mechanism can infuse the gap between cellular and tissue level strains, it was derived using idealized models of osteocyte process and canaliculus, regardless of their irregular shapes (Kamioka et al. 2012). The high-resolution image-based model (Fig. 1) developed using UHVEM tomography (Kamioka et al. 2012; Hosaki-Takamiya et al. 2016) was utilized for conducting fluid–structure interaction simulations. This approach facilitated the evaluations of the distribution and magnitude of local strain on the osteocyte process, considering the heterogeneous inclination of TEs.

The image-based fluid–structure interaction simulation results reveal that a small number of TEs with high tension induced local strain concentrations that were heterogeneously distributed in the osteocyte process. These findings were commonly observed regardless of the variation in TE configurations, suggesting that TE-induced strain concentration in the osteocyte process is a universal feature associated with osteocyte mechanosensing. In addition, mechanotransductive ion channels on the cell membrane, such as Piezo1, are considered important for osteocyte mechanosensing as they respond to the neighboring membrane strain (McMahon et al. 2008; Li et al. 2019; Diem et al. 2020; Sasaki et al. 2020; Zhou et al. 2020). Thus, the locally concentrated strain on the osteocyte process, which was observed in this study, can cause structural changes in the ion channels as an activation of mechanosensing. Application of local deformation has been demonstrated to induce calcium transients in the vicinity of the stimulated point and cause diffusive wave propagation of the calcium transient to the entire intracellular region (Adachi et al. 2009). Moreover, the specific TEs contributing to the osteocyte strain concentration, which appeared to be inclined toward the upstream interstitial fluid flow in the osteocyte process (Fig. 4), were determined based on the irregular shape of osteocyte processes and canaliculi (Fig. 1b). Therefore, the transformation of the surface structure pertaining to the osteocyte process and canaliculus can considerably influence the distribution and magnitude of the osteocyte strain. This signifies that bone diseases such as osteoporosis that are accompanied by alterations in the lacuna-canalicular structure (Knothe Tate et al. 2004) are closely associated with the abnormal mechanical stimuli transmitted to osteocytes owing to the variation in the distribution of TEs in canaliculi.

One of the limitations of this analysis is that TEs were assumed to be randomly arranged in the canaliculi owing to lack of information regarding their distribution. Thus, future development of imaging technology would enable visualization of the osteocyte-specific distribution of TEs in canaliculi. In context, the image-based fluid–structure interaction simulation considering actual TE distribution will help identify the molecules responsible for osteocyte mechanosensing. This can be achieved by comparing the obtained strain distribution with the location of candidate molecules, which can be detected using techniques such as structured illumination microscopy-based super-resolution microscopy (Kiuchi et al. 2015; Cabahug-Zuckerman et al. 2018). An additional limitation of the present analysis is the restricted size of the analyzed region in relation to the canalicular network scale, which originates from the limitation posed by the electron transmissibility of UHVEM. In a bone tissue, numerous osteocytes form a complex network via their branching processes, which can change the flow velocity inside each canaliculus (van Tol et al. 2020a; b). Therefore, the strain concentration regions in the osteocyte network can be clarified with an analysis considering the network structure. In context, focused ion beam-scanning electron microscopy (FIB-SEM) is a possible solution for visualizing the network structure at a high resolution (Hashimoto et al. 2017; Hasegawa et al. 2018; Hirashima et al. 2020). Fluid–structure interaction simulation using the FIB-SEM image-based model will enable the evaluation of the impact of the network structure on mechanical stimuli to osteocytes.

In summary, results of the simulation conducted in this study reveal that local strain concentration in the osteocyte process was induced by a small number of TEs with high tension, which are determined by the irregular shapes of osteocyte processes and canaliculi. These results provide meaningful insights into the effect of the ultrastructure of osteocyte processes and canaliculi on osteocyte mechanical stimuli. We anticipate that this study will help in revealing molecular mechanisms that activate osteocyte mechanosensing and understanding the regulatory mechanisms of bone remodeling.

## Data Availability

All data needed to evaluate the conclusions of this study are presented herein. Additional data related to this study may be requested from the authors.

## Code availability

Source codes related to this study may be requested from the authors.
